# Management of Intraoperative Erection in Endourology: A Systematic Review of Techniques and Interventions

**DOI:** 10.7759/cureus.99000

**Published:** 2025-12-11

**Authors:** Faiz A Shaikh, Daneyal Arshad, Jas Kalsi

**Affiliations:** 1 Urology, Wexham Park Hospital, Frimley Health NHS Foundation Trust, Slough, GBR

**Keywords:** endourology and other urology specialitues, intracavernosal injection, intraoperative erection, sympathomimetics, systematic literature review

## Abstract

Intraoperative penile erection is an uncommon but significant complication encountered during endourological procedures, such as Transurethral Resection of the Prostate (TURP), Transurethral Resection of the Bladder Tumour (TURBT), and ureteroscopy, with incidence rates varying by anaesthetic technique. This phenomenon can impede surgical access, compromise patient safety, and increase the risk of urethral injury and long-term sequelae. Existing practice for its management is shaped by limited evidence and lacks standardised protocols. This review aims to systematically examine the literature on management strategies for intraoperative erection during endourological surgery and to evaluate the efficacy and safety of various interventions, providing evidence-based recommendations for clinical management.

The review adhered to Preferred Reporting Items for Systematic Reviews and Meta-Analyses (PRISMA) guidelines, analysing studies from PubMed, EMBASE®, and the Cochrane Library. Search terms included “intraoperative erection” and related phrases. Eligible studies addressed pharmacological or non-pharmacological management of intraoperative erection, excluding those on erectile dysfunction, non-human studies, or non-English articles. Two independent assessors reviewed studies to minimise bias, and quality appraisal was performed using the JBI tool and Oxford Centre for Evidence-Based Medicine grading.

Nineteen studies reporting management of intraoperative erection in more than 122 cases over a 40-year period (1983-2023) were included. The majority were case series (n=12) and letters to the editor (n=4), with three case reports. Intracavernosal sympathomimetic agents, particularly phenylephrine (93-100% success rate), ephedrine (100% success rate), and epinephrine (100% success rate), demonstrated the highest efficacy with rapid detumescence (1-5 minutes) and minimal complications. Intravenous sympathomimetics showed variable success rates, with terbutaline achieving 100% success but causing systemic side effects, while ketamine demonstrated inconsistent results (0-78%). Alternative approaches, including dorsal nerve block and cold saline compresses, were effective but less commonly reported.

The findings underscore the lack of robust, high-quality evidence for managing intraoperative erection, with current strategies being largely anecdotal or adapted from other contexts. While intracavernosal injection of sympathomimetic agents, particularly phenylephrine, is the most effective first-line management based on available evidence, this is predominantly supported by low-level data (Oxford Level 4-5). The review highlights the urgent need for comprehensive prospective studies, especially randomised controlled trials, to establish standardised, evidence-based treatment protocols. Given the clinical significance of this complication, it also warrants greater attention, including explicit inclusion in preoperative consenting discussions.

## Introduction and background

Intraoperative erection, a full, sustained penile erection occurring after the induction of anaesthesia, is an uncommon yet problematic complication that can occur during endourological procedures, including Transurethral Resection of the Prostate (TURP), Transurethral Resection of Bladder Tumour (TURBT), and ureteroscopy. The reported incidence varies depending on the type of anaesthesia used, ranging from 0.34%-3.5% during general anaesthesia and 0.11%-0.34% for spinal anaesthesia [1-3].

The clinical significance of intraoperative erection extends beyond mere surgical inconvenience. During transurethral procedures, penile tumescence prevents adequate passage of endoscopes and surgical instruments through the urethra, significantly interfering with surgical access and impairing visualisation of the surgical site. This can compromise the surgeon’s ability to perform the intended operation safely and effectively, potentially leading to procedural delays, the need for repeat procedures, increased risk of urethral injury, including acute complications such as bleeding, and long-term sequelae such as urethral stricture formation [1].

The pathophysiology of intraoperative erection remains poorly understood but involves autonomic imbalance during anaesthesia, with suppression of sympathetic (anti-erectile) tone and preserved or stimulated parasympathetic pathways, thus promoting penile tumescence [2].

Various treatment modalities have been proposed, ranging from conservative measures such as observation, to pharmacological interventions including intravenous and intracavernosal injections of sympathomimetic drugs, and physical manoeuvres such as intracavernosal aspiration [4-8]. Many of these approaches overlap with strategies used to manage priapism, leveraging similar pathophysiological principles of penile detumescence.

However, there is limited high-quality evidence, such as randomised controlled trials or comparative studies, to guide management decisions intraoperatively. The lack of standardised management protocols and the predominance of case reports and small case series in the literature underscore the need for a comprehensive synthesis of available evidence.

This systematic review aims to identify, synthesise, and evaluate all published evidence on intraoperative erection management during endourological surgery, assessing intervention efficacy, safety profiles, time to detumescence, and providing evidence-based clinical recommendations.

To clearly structure the research question, the PICO framework was utilised. The population examined includes patients undergoing endourological surgery who develop intraoperative erection. Interventions under consideration comprised both pharmacological and non-pharmacological methods aimed at detumescence. Comparisons were made between the different treatment approaches identified in the literature. The outcomes assessed included the success rate of detumescence, the time required to achieve detumescence, the ability to continue with the surgical procedure, and the incidence of any adverse events. This structure ensures a focused and methodical synthesis of the current evidence, ultimately aiming to support clinical practice with robust, evidence-based guidance.

## Review

Materials and methods

This systematic review was conducted in accordance with the Preferred Reporting Items for Systematic Reviews and Meta-Analyses (PRISMA) 2020 guidelines. The review was designed to transparently report the methodology, data sources, selection criteria, and analytical approach used to synthesise the available evidence [9].

Information Sources and Search Strategy

A comprehensive literature search was performed to identify all relevant studies on the management of intraoperative erection. Three major electronic databases, PubMed, EMBASE, and Cochrane (reviews and trials), were systematically searched. Additional sources, including reference lists of relevant articles, were also screened to identify studies not captured in the primary database search.

Search terms included: “intraoperative erection,” “management,” “sympathomimetics,” “endourological surgery,” and related keywords.

Eligibility Criteria

Inclusion required studies reporting management of intraoperative erection during endourological procedures in humans. All types of study designs were considered, encompassing randomised controlled trials, observational studies, case series, case reports, and expert opinions or letters to the editor. Only studies written in English or those with accessible English translations were included.

Studies were excluded if they focussed on non-intraoperative priapism or erectile dysfunction, as were studies that investigated only the prevention or incidence of intraoperative erection without addressing management strategies. Non-human or in vitro studies were also omitted from consideration. Furthermore, articles for which the full text could not be retrieved, despite efforts to contact authors or institutional libraries, were excluded. Lastly, studies published in non-English languages without accessible translations were not included.

Screening and Selection Process

The screening process was conducted in two stages following PRISMA guidelines.

An initial screening of titles and abstracts of all unique records was performed to identify potentially relevant articles. Articles clearly not meeting the above inclusion criteria were excluded at this stage.

Full-text articles were then sought for the potentially eligible records, and these underwent detailed evaluation against the eligibility criteria.

Data Extraction

A structured data extraction form was created to ensure systematic collection of relevant information from each study included in the review. This form captured key study characteristics, such as the names of the authors, year of publication, and study design. Patient demographics were also recorded, including the number of cases reported, the age range of participants, and any relevant co-morbidities. For each intervention, details were documented regarding the specific treatment modality employed, the route of administration, drug dosage, and the timing of the intervention. In terms of outcomes, data were collected on the success rate of detumescence, the time required to achieve detumescence, the ability to continue the surgical procedure, and the occurrence of any adverse events or complications.

Quality Assessment

Included studies were appraised using the Joanna Briggs Institute (JBI) critical appraisal tools tailored to each study design. These tools are publicly available and have been methodologically detailed in prior publications to ensure rigorous assessment of study quality and bias [10, 11].

In addition, each study was classified according to the Oxford Centre for Evidence-Based Medicine classification [12, 13]. The collected data were tabulated to allow comparison of success rates, time to detumescence, and complication profiles across different treatment modalities.

Data Synthesis

Due to extreme heterogeneity (study designs, dosing, populations) and low-level evidence (Oxford 4-5), formal meta-analysis was not performed. Descriptive synthesis with narrative summaries and tables was used to identify consistent patterns across interventions.

Results

**Figure 1:**
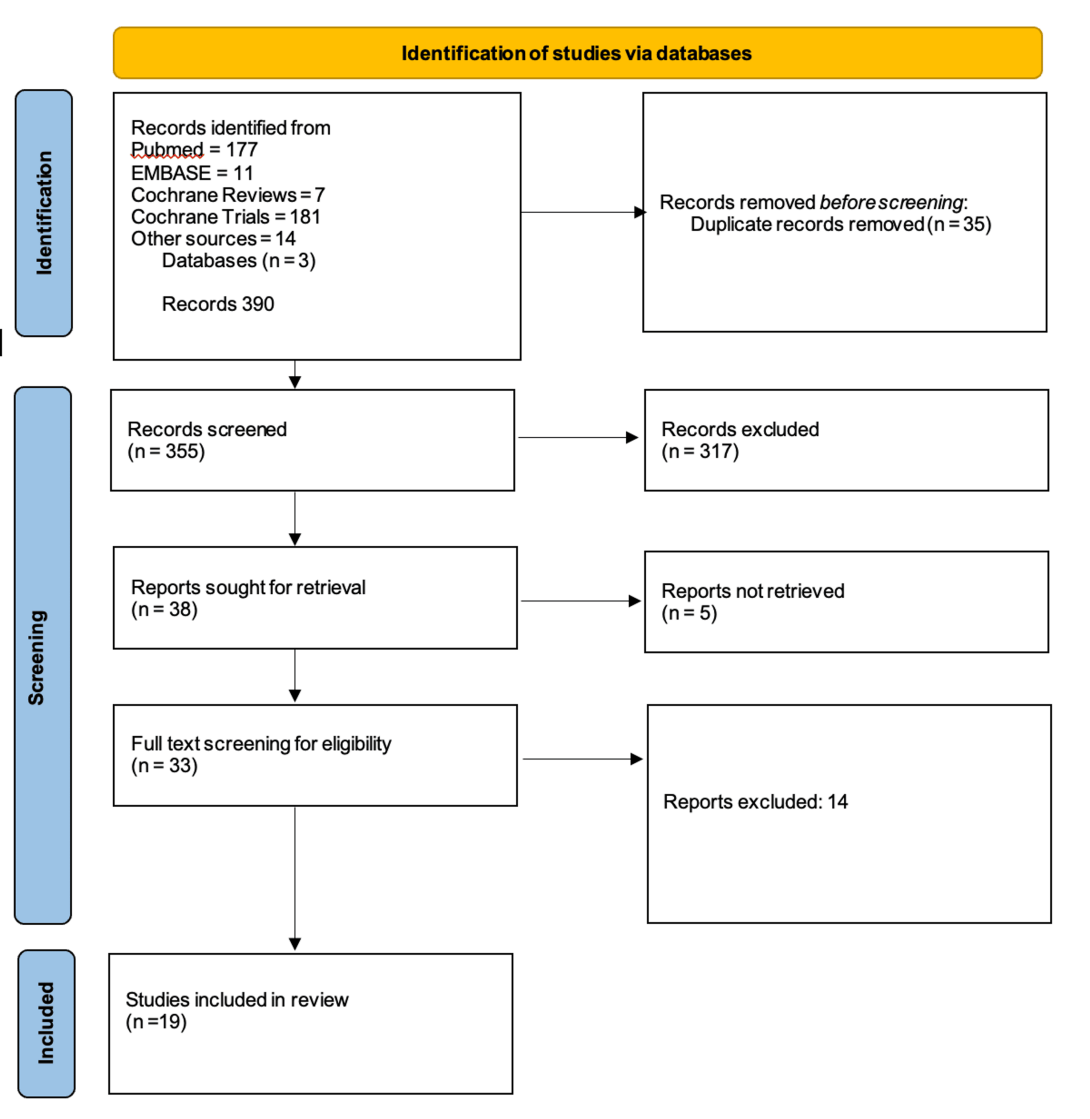
Preferred Reporting Items for Systematic Reviews and Meta-Analyses (PRISMA) flow diagram for the included database searches.

The systematic search identified 390 records from electronic databases and other sources. After removal of 35 duplicates, 355 records underwent title and abstract screening, resulting in exclusion of 317 records that did not meet inclusion criteria. Full-text articles were sought for 38 potentially relevant studies, of which 5 could not be retrieved. Following detailed full-text review of 33 articles, 14 were excluded. A total of 19 studies met all eligibility criteria and were included in the systematic review (Figure 1).

The 19 included studies were published between 1983 and 2023, spanning four decades of clinical experience. The study designs comprised 12 case series (63%), 4 letters to the editor or expert opinions (21%), and 3 case reports (16%). Collectively, these studies reported management of intraoperative erection in more than 122 individual cases, though the exact total could not be precisely determined, as one study did not report the specific number of cases (Table 1) [1].

**Table 1:** Summary of all 19 included studies. * This study has been included twice as it investigated two different treatment modalities. ** Initial treatment failure with intravenous glycopyrrolate followed by successful treatment with dorsal penile nerve block.

Author and year	Study type	Number of cases	Treatments	Success rate	Complications	Oxford level of evidence
van Arsdalen KN et al. (1983) [14]	Case series	3	Intravenous ketamine (20-200 mg)	0%	Abandonment of procedure	4
Sundien E and Kolmert T (1987) [15]	Letter to the editor	4	Intracavernosal aspiration	100%	Continuous dripping of blood from needle	5
Sundien E and Kolmert T (1987) [15]*	Letter to the editor	30	Intracavernosal ephedrine (5 mg)	100% (within 2 minutes)	Nil reported	5
Walther PJ et al. (1987) [4]	Case series	3	Intracavernosal phenylephrine (0.1 mg)	100% (within 1-2 minutes)	Nil reported	5
Miyabe M and Namiki A (1988) [16]	Letter to the editor	1	Intravenous ephedrine (10 mg + 10 mg + 20 mg)	100% (within 5 minutes)	Nil reported	5
Shantha TR (1989) [17]	Case series	4	Intravenous terbutaline (0.25-0.5 mg)	100% (within 4-5 minutes)	Tachycardia and hypertension	4
Tsai SK and Hong CY (1990) [5]	Case series	5	Intravenous metaraminol (0.1-0.25 mg)	100% (within 2 minutes)	Nil reported	4
Serrate RG et al. (1992) [6]	Case series	15	Intravenous ethylphrine (10 mg)	100% (within 2-4 minutes)	Nil reported	4
Seftel AD et al. (1994) [18]	Case report	1	Dorsal nerve block (5 mL of 1:1 1% lidocaine + 0.5% bupivacaine)	100% (*immediate)	Nil reported	4
Staerman et al. 1995 [7]	Case series	23	Intracavernosal phenylephrine (0.2 mg)	100% (within 2-3 minutes)	Local haematoma (n=1), transient rise in BP ≈30 mmHg (n=3)	4
Gerber F et al. (2001) [19]	Letter to the editor	1	Intracavernosal epinephrine (0.001 mg)	100% (within 2 minutes)	Nil reported	5
Rao TH et al. (2001) [20]	Case series	2	Cold saline compress + intravenous terbutaline (0.5 mg)	100% (30-45 minutes)	Nil reported	4
Rao TH et al. (2001) [20]*	Case series	1	Cold saline compress + intravenous glycopyrrolate (0.2 mg)	100% (within 30 minutes)	Nil reported	4
Brierly RD et al. (2003) [21]	Case report	5	Intracavernosal lidocaine (44 mg lidocaine + 0.0257 mg epinephrine)	100% (within 2 minutes)	Nil reported	4
Baltogiannis DM et al. (2006) [22]	Case series	3	Intracavernosal phenylephrine (0.25 mg) + cold saline compress	66.6% (2-20 minutes)	Nil reported	4
Guler G et al. (2012) [23]	Case series	12	Intravenous dexmedetomidine (0.5 μg/kg)	83% (within 9 minutes)	Nil reported	4
Prakash S et al. (2012) [24]	Letter to the editor	1	Inhaled salbutamol via endotracheal tube (total dose 1.6 mg)	100% (within 20 minutes)	Nil reported	5
Gray M et al. (2017) [1]	Case series	Not reported	Intracavernosal ephedrine (15 mg)	100%	Minor bruising	4
Eroğlu A et al. (2020) [25]	Case series	9	Intravenous ketamine (18-75 mg)	77.80%	Nil reported	5
Yadav NK et al. (2021) [8]	Case report	1	Intracavernosal ephedrine (15 mg)	100% (within 1 minute)	Nil reported	4
Hoda W et al. (2023) [3]	Case series	1	Intracavernosal epinephrine (0.2 mg)	100% (within 5 minutes)	Nil reported	4
Hoda W et al. (2023) [3]*	Case series	1	Intravenous glycopyrrolate (0.2 mg) followed by dorsal penile nerve block (80 mg levobupivacaine)	100% (within 10 minutes)**	Nil reported	4

The studies originated from diverse geographical regions, including the United States, Europe, and Asia, reflecting the global recognition of this clinical challenge. All studies involved male patients undergoing various endourological procedures, most commonly TURP, TURBT, and retrograde intrarenal surgery. Both general and regional anaesthesia were represented across the included studies.

All the studies included were categorised based on the Oxford Centre for Evidence-Based Medicine Levels of Evidence. Of the 19 studies reviewed, 12 were classified as Level 4 evidence, consisting of case series and case reports that applied clear, systematic methods. The other 7 were considered Level 5 evidence, including expert opinions and letters to the editor that used limited systematic methodologies.

Quality appraisal using JBI tools revealed variable methodological rigour across studies, consistent with their low levels of evidence.

No randomised controlled trials, cohort studies, or case-control studies specifically evaluating treatment of established intraoperative erection were identified, reflecting the rarity of this condition and the ethical and practical challenges of conducting prospective comparative studies.

Case reports [8, 18, 21] generally provided clear descriptions of interventions and outcomes, with all offering clinically relevant takeaway lessons. However, patient demographic characteristics were poorly reported in most cases, and post-intervention clinical conditions were often described in an unclear manner. The temporal sequence of events was best described in the most recent report by Yadav NK et al. (Table 2) [8].

**Table 2:** The Joanna Briggs Institute (JBI) critical appraisal tool for use in JBI systematic reviews: checklist for case reports.

Study ID	Seftel AD et al. (1994) [18]	Brierly RD et al. (2003) [21]	Yadav NK et al. (2021) [8]
Were the patient's demographic characteristics clearly described?	No	No	No
Was the patient's history clearly described and presented as a timeline?	Unclear	No	Yes
Was the current clinical condition of the patient on presentation clearly described?	Yes	Unclear	Yes
Were diagnostic tests or assessment methods and the results clearly described?	N/A	N/A	N/A
Was the intervention(s) or treatment procedure(s) clearly described?	Yes	Yes	Yes
Was the post-intervention clinical condition clearly described?	Unclear	Unclear	Unclear
Were adverse events (harms) or unanticipated events identified and described?	N/A	N/A	N/A
Does the case report provide takeaway lessons?	Yes	Yes	Yes

Case series demonstrated considerable variability in quality [1, 3-7, 14, 17, 20, 22, 23, 25]. Most studies (10/12) clearly defined inclusion criteria for cases. However, consecutive patient inclusion was unclear or absent in the majority (7/12), potentially introducing selection bias. Demographic and clinical information was inadequately reported in most series, with only three studies (Hoda W et al., Baltogiannis DM et al., Eroğlu A et al.) providing satisfactory clinical details [3, 22, 25]. Outcome reporting was generally adequate, though follow-up information was limited or unclear in several studies (Table 3).

**Table 3:** The Joanna Briggs Institute (JBI) critical appraisal tool for use in JBI systematic reviews: checklist for case series.

Study ID	van Arsdalen KN et al. (1983) [14]	Walther PJ et al. (1987) [4]	Shantha TR (1989) [17]	Tsai SK et al. (1990) [5]	Serrate RG et al. (1992) [6]	Staerman F et al. (1995) [7]	Rao TH et al. (2001) [20]	Baltogiannis DM et al. (2006) [22]	Guler G et al. (2012) [23]	Gray M et al. (2017) [1]	Eroğlu A et al. (2020) [25]	Hoda W et al. (2022) [3]
Were there clear criteria for inclusion in the case series?	Yes	No	Unclear	Yes	Yes	Yes	Yes	Yes	Yes	Yes	Yes	Yes
Was the condition measured in a standard, reliable way for all participants included in the case series?	N/A	N/A	N/A	N/A	N/A	N/A	N/A	N/A	N/A	N/A	N/A	N/A
Were valid methods used for identification of the condition for all participants included in the case series?	N/A	N/A	N/A	N/A	N/A	N/A	N/A	N/A	N/A	N/A	N/A	N/A
Did the case series have consecutive inclusion of participants?	No	No	No	No	No	Yes	Unclear	Yes	Yes	Unclear	Yes	No
Was there clear reporting of the demographics of the participants in the study?	Yes	Unclear	No	No	No	Unclear	No	No	No	No	Unclear	No
Was there clear reporting of clinical information of the participants?	Yes	Unclear	Unclear	No	No	No	No	Yes	Unclear	No	Yes	Yes
Were the outcomes of follow-up or results of cases clearly reported?	Yes	Yes	Yes	Yes	Unclear	Yes	Unclear	Yes	Unclear	No	Yes	Yes
Was there clear reporting of the presenting sites/clinic demographic information?	Unclear	Unclear	No	No	No	Unclear	No	No	Unclear	Unclear	Yes	Unclear
Was statistical analysis appropriate?	N/A	N/A	N/A	N/A	N/A	N/A	N/A	N/A	N/A	N/A	N/A	N/A

Expert opinions and letters to the editor showed reasonable quality for their evidence level [15,16,19,24]. Most demonstrated logical arguments supporting their conclusions and referenced existing literature appropriately [19,24]. However, identification of opinion sources and their credentials was often unclear, particularly in earlier publications (Table 4) [15,16].

**Table 4:** The Joanna Briggs Institute (JBI) critical appraisal tool for use in JBI systematic reviews: checklist for expert opinion.

Study ID	Sundien E et al. (1987) [15]	Miyabe M and Namiki A (1988) [16]	Gerber F et al. (2001) [19]	Prakash S et al. (2012) [24]
Is the source of the opinion clearly identified?	Unclear	Unclear	Unclear	Yes
Does the source of opinion have standing in the field of expertise?	Unclear	Unclear	Unclear	Yes
Are the interests of the relevant population the central focus of the opinion?	Yes	Unclear	Yes	Yes
Does the opinion demonstrate a logically defended argument to support the conclusions drawn?	Yes	Yes	Yes	Yes
Is there reference to the extant literature?	No	No	Yes	Yes
Is any incongruence with the literature/sources logically defended?	Unclear	Yes	Yes	Yes

Treatment Modalities and Outcomes: Intracavernosal Injection of Sympathomimetic Agents

Intracavernosal injection of sympathomimetic agents emerged as the most frequently reported and effective intervention for intraoperative erection management.

Intracavernosal phenylephrine was evaluated in three studies involving 26 patients [4, 7, 22]. Walther PJ et al. (1987) reported successful detumescence in 3 patients using 0.1 mg phenylephrine, with resolution occurring within 1-2 minutes and no complications [4]. Staerman F et al. (1995) presented the largest series, treating 23 cases with 0.2 mg intracavernosal phenylephrine [7].

All cases achieved detumescence within 2-3 minutes, with minimal complications, one local haematoma and three cases of transient blood pressure elevation (approximately 30 mmHg increase). Baltogiannis DM et al. (2006) combined intracavernosal phenylephrine (0.25 mg) with cold saline compresses in 3 patients, achieving success in 2 cases (66.6%), with detumescence occurring between 2-20 minutes and no reported complications [22].

Intracavernosal phenylephrine achieved success rates of 93-100% with rapid onset of action (1-3 minutes). There was an acceptable safety profile characterised by infrequent minor local complications and occasional transient systemic effects.

Intracavernosal ephedrine was reported in three studies with excellent outcomes [1, 8, 15]. Sundien E and Kolmert T (1987) described 30 cases treated with 5 mg intracavernosal ephedrine, achieving 100% success with detumescence within 2 minutes, though continuous dripping of blood from the needle site was noted [15]. Gray M et al. (2017) reported universal success with 15 mg intracavernosal ephedrine, with only minor bruising as a complication [1]. Yadav NK et al. (2021) successfully managed one case with 15 mg intracavernosal ephedrine, achieving detumescence within 1 minute without complications [8].

Intracavernosal ephedrine demonstrated 100% efficacy across all reported cases, with rapid action (1-2 minutes) and minimal complications limited to minor local effects such as bruising and needle-site bleeding.

Intracavernosal epinephrine was evaluated in two studies involving 3 patients total [3, 19]. Gerber F et al. (2001) reported successful treatment of one case using a very low dose of 0.001 mg (1 microgram) epinephrine, with detumescence achieved within 2 minutes and no complications [19]. Hoda W et al. (2023) successfully treated 2 cases with 0.2 mg intracavernosal epinephrine, achieving detumescence within 5 minutes without adverse events [3]. Additionally, Brierly RD et al. (2003) used intracavernosal lidocaine (44 mg) combined with epinephrine (0.0257 mg) in one patient, achieving resolution within 2 minutes without complications [21].

The limited available data on intracavernosal epinephrine suggest 100% efficacy with rapid detumescence (2-5 minutes) and no reported complications, though the evidence base remains small.

Treatment Modalities and Outcomes: Intravenous Sympathomimetic Agents

Several intravenous sympathomimetic medications have been investigated as alternatives to intracavernosal injection.

Miyabe M and Namiki A (1988) reported one case managed with escalating doses of intravenous ephedrine (10 mg, then 10 mg, then 20 mg for a total of 40 mg), achieving 100% success with detumescence within 5 minutes and no complications [16].

Two studies evaluated intravenous terbutaline [17, 20]. Shantha TR (1989) treated 4 patients with doses ranging from 0.25-0.5 mg, achieving 100% success with detumescence within 4-5 minutes, though significant complications, including tachycardia and hypertension, were observed [17]. Rao TH et al. (2001) used intravenous terbutaline (0.5 mg) as part of a combination approach with cold saline compresses and intravenous glycopyrrolate (0.2 mg) in 3 patients, achieving 100% success, though timing was not clearly reported [20].

While intravenous terbutaline demonstrated 100% efficacy, its use was associated with systemic cardiovascular side effects, which must be considered if selecting this intervention.

Tsai SK and Hong CY (1990) reported 100% success in 5 patients treated with intravenous metaraminol (0.1-0.25 mg), achieving detumescence within 2 minutes without complications [5]. Serrate RG et al. (1992) described the largest series of intravenous sympathomimetic use, treating 15 patients with 10 mg intravenous ethylphrine and achieving 100% success within 2-4 minutes without reported adverse events [6].

Treatment Modalities and Outcomes: Local/Regional Anaesthetic Techniques

Dorsal penile nerve block represented an alternative non-sympathomimetic approach to managing intraoperative erection.

Seftel AD et al. (1994) reported immediate resolution of intraoperative erection following dorsal nerve block using 5 mL of a 1:1 mixture of 1% lidocaine and 0.5% bupivacaine, with no complications [18]. Hoda W et al. (2023) successfully used dorsal nerve block with 80 mg levobupivacaine in one patient who had failed to respond to intravenous glycopyrrolate, achieving detumescence within 10 minutes without adverse events [3].

The dorsal nerve block technique demonstrated 100% success in the limited reported cases (n=3), with rapid to moderate onset (immediate to 10 minutes) and no complications.

Treatment Modalities and Outcomes: Physical and Non-Pharmacological Methods

Application of cold saline-soaked sponges or ice packs to the penis was evaluated in two studies [20,22]. Rao TH et al. (2001) used cold saline compresses as part of a combination approach with intravenous medications in 3 patients, achieving 100% success, though timing was unclear [20]. Baltogiannis DM et al. (2006) combined cold saline compresses with intracavernosal phenylephrine in 3 patients, achieving 66.6% success with variable timing (2-20 minutes) [22].

Cold saline compresses represent a simple, non-invasive intervention that may be useful as an adjunct or initial temporising measure, though efficacy appears variable and time to effect can be prolonged.

Sundien E and Kolmert T (1987) reported attempting intracavernosal aspiration of blood in 4 patients, achieving 100% success, though continuous dripping from the needle site was noted as a complication [15]. This mechanical approach may be considered when pharmacological methods are unavailable, though it was subsequently superseded by intracavernosal sympathomimetic injection in the same report.

Treatment Modalities and Outcomes: Alternative Approaches

Guler G et al. (2012) evaluated intravenous dexmedetomidine (0.5 μg/kg) in 12 patients, reporting an 83% success rate, with detumescence achieved within a mean of 9 minutes in responders and no complications [23]. The study reported an institutional incidence of intraoperative erection of 0.34% during general anaesthesia, 0.11% during spinal anaesthesia, and 1.72% during epidural anaesthesia. Two patients (17%) did not respond to dexmedetomidine within 15 minutes.

Intravenous ketamine produced inconsistent results across two studies [14,25]. van Arsdalen KN et al. (1983) reported complete failure (0% success rate) in 3 patients treated with intravenous ketamine [14]. Conversely, Eroğlu A et al. (2020) achieved 77.8% success in 9 patients using doses ranging from 18-75 mg, though timing of response was not reported [25]. This marked discrepancy in outcomes makes ketamine an unreliable choice for management of intraoperative erection.

Glycopyrrolate, a muscarinic antagonist, was evaluated in two studies with contrasting results [3, 20]. Rao TH et al. (2001) used it as part of a combination regimen (with terbutaline and cold compresses) with apparent success [20]. However, Hoda W et al. (2023) reported that intravenous glycopyrrolate (0.2 mg) alone failed to achieve detumescence (0% success) [3].

Prakash S et al. (2012) described a unique case managed with inhaled salbutamol (total dose 1.6 mg) administered via endotracheal tube during general anaesthesia, achieving 100% success within 20 minutes without complications [24]. While successful, this approach represents the longest time to detumescence among pharmacological interventions and has not been replicated in subsequent reports.

Comparative Analysis of Treatment Modalities

Synthesis of the evidence reveals clear patterns in treatment efficacy, speed of action, and safety profiles across different intervention categories. Efficacy: Intracavernosal sympathomimetics (phenylephrine, ephedrine, epinephrine) demonstrated the highest and most consistent success rates (93-100%), followed by select intravenous sympathomimetics such as metaraminol and ethylphrine (100%) and terbutaline (100%). Dorsal nerve block also achieved 100% success in limited cases. Less reliable interventions included intravenous dexmedetomidine (83%), cold saline compresses (67-100%), and especially intravenous ketamine (0-78%).

Speed of detumescence: Intracavernosal sympathomimetics produced the fastest responses, with most cases achieving detumescence within 1-5 minutes. Intravenous sympathomimetics (excluding ketamine) typically acted within 2-9 minutes. Dorsal nerve block varied from immediate to 10 minutes. Physical methods such as cold saline compresses showed the most variable timing (2-20 minutes), and inhaled salbutamol required 20 minutes.

Safety profile: Intracavernosal sympathomimetics were generally well tolerated, with only minor local complications (occasional haematoma, bruising, transient blood pressure elevation) in a small minority of cases. Intravenous terbutaline was associated with systemic cardiovascular effects, including tachycardia and hypertension. Other intravenous agents, dorsal nerve block, and physical methods showed favourable safety profiles with no or minimal reported complications.

Practical considerations: Intracavernosal injection requires familiarity with penile anatomy and injection technique but can be performed rapidly at the surgical site. Intravenous administration may be preferred when vascular access is already established, though systemic effects are a consideration. Dorsal nerve block requires regional anaesthetic expertise. Physical methods are universally accessible but may have limited efficacy as monotherapy.

Discussion

Summary of Findings

This systematic review synthesised evidence from 19 studies reporting management of intraoperative erection in more than 122 cases over a 40-year period. The findings demonstrate that intracavernosal sympathomimetic agents, particularly phenylephrine, represent the most effective treatment modality with acceptable safety profiles characterised by infrequent minor complications. Intravenous sympathomimetic agents showed variable efficacy, with only terbutaline causing systemic cardiovascular side effects. Alternative approaches including dorsal nerve block and cold saline compresses were effective in limited cases but less commonly employed.

The evidence base consists entirely of case reports, case series, and expert opinions (Oxford Level 4-5 evidence), reflecting the rarity of this complication and the practical challenges of conducting prospective comparative studies. Despite methodological limitations, the consistency of outcomes across multiple independent reports from diverse geographical locations strengthens confidence in the overall findings.

Implications for Practice

Although the mechanisms behind intraoperative erection are not fully understood, knowledge of physiological erection can inform management and guide effective intervention for this complication. Penile erection is a neurovascular process, governed by a coordinated interplay between the sympathetic, parasympathetic, and somatic nervous systems [26]. Therefore, this can be precipitated by autonomic imbalance during anaesthesia, namely, suppression of sympathetic tone and preservation or stimulation of parasympathetic pathways [2]. As a result, intraoperative erections may develop, complicating surgical procedures and necessitating intervention.

The foundation for the superior efficacy of intracavernosal sympathomimetic agents in treating these erections can be explained by this physiological interplay: direct injection of alpha-adrenergic agonists such as phenylephrine, ephedrine, and epinephrine swiftly restores sympathetic vasoconstriction at the target tissue, counteracting anaesthesia-induced shifts in autonomic balance and facilitating detumescence.

Dexmedetomidine exhibits a distinct mechanism of action compared to traditional sympathomimetics used intraoperatively for penile detumescence. As a highly selective alpha-2 adrenergic receptor agonist, dexmedetomidine centrally reduces sympathetic outflow by inhibiting norepinephrine release, leading to sedation and analgesia without directly stimulating adrenergic receptors peripherally [27]. Although penile erection is primarily a parasympathetic-mediated process, dexmedetomidine’s sympatholytic effects restore autonomic balance and promote detumescence, likely through central modulation and peripheral vasoconstriction mediated by alpha-2 receptors on vascular smooth muscle. This contrasts with sympathomimetics, which induce detumescence via direct alpha-1 adrenergic receptor stimulation causing vasoconstriction [26]. Dexmedetomidine’s unique pharmacologic profile offers advantages in patients where sympathomimetics are contraindicated, such as those with cardiovascular disease or predisposition to arrhythmias, or when sympathomimetics prove ineffective. Its sedative properties and reduced cardiovascular side effects make it a valuable alternative in managing intraoperative penile erection, with reported success rates exceeding 80% [23].

Psychogenic stimulation from dreams or increased sensory processing under anaesthesia may also contribute to intraoperative erection, especially during direct penile manipulation by the surgeon [28]. A dorsal penile nerve block offers a non-adrenergic pharmacological solution to reduce physical stimulation. This technique is usually carried out by trained clinicians, such as urologists.

Considering the range of available modalities and the fact that patients are usually not consented for intracavernosal procedures during the pre-operative process for endourological surgeries, it can be challenging to determine the best initial approach if an intraoperative erection occurs.

**Figure 2:**
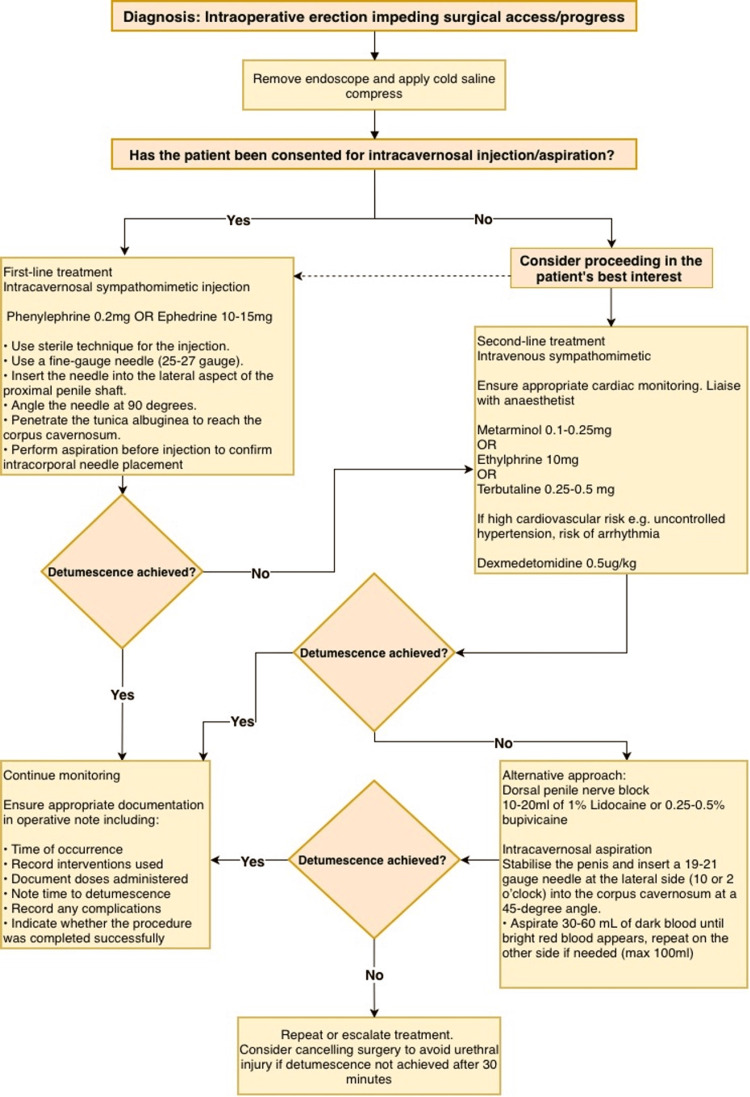
Suggested management algorithm for intraoperative erection. Figure created by the authors based on data from this systematic review.

By synthesising the evidence presented in this paper, the following recommendation can be made for clinical practice (Figure 2):

First-Line Management

Intracavernosal injection of sympathomimetic agents should be considered the first-line treatment for intraoperative erection. Phenylephrine (0.1-0.25 mg), ephedrine (5-15 mg), or epinephrine (0.001-0.2 mg) injected directly into the corpus cavernosum offer the highest success rates (93-100%), most rapid onset (1-5 minutes), and acceptable safety profiles. The EAU priapism guidelines recommend intracavernosal phenylephrine at 200 μg (0.2 mg) aliquots every 3-5 minutes, up to a maximum of 1 mg within 1 hour [29]. While these doses can be repeated for intraoperative erection, the lower starting dose (0.1 mg) is reasonable in the anaesthetised patient, as general and spinal anaesthesia can alter vascular responsiveness and systemic absorption compared with awake patients. Phenylephrine may be preferred as the most extensively studied agent with the largest evidence base, although ephedrine or epinephrine can be used where phenylephrine is unavailable.

Technique for Intracavernosal Injection

The injection should be performed using sterile technique with a fine-gauge needle (25-27 gauge) inserted into the lateral aspect of the proximal penile shaft at a 90-degree angle, penetrating the tunica albuginea to deliver medication directly into the corpus cavernosum. Aspiration prior to injection confirms intracorporal placement. Given the vascular communication between the corpora cavernosa, unilateral injection typically suffices [30]. Patients should be monitored for cardiovascular changes, though systemic absorption is minimal at recommended doses.

Alternative Options

When intracavernosal injection is not feasible or contraindicated, intravenous sympathomimetics (metaraminol 0.1-0.25 mg, terbutaline 0.25-0.5 mg, dexmedetomidine 0.5 μg/kg) represent reasonable alternatives, with awareness of potential systemic side effects, particularly with terbutaline. Dorsal penile nerve block offers an effective non-adrenergic option when performed by clinicians experienced in regional anaesthetic techniques.

Adjunctive Measures

Cold saline compresses may be applied as a temporising measure while preparing pharmacological interventions or as an adjunct to hasten response, though they should not be relied upon as monotherapy given variable efficacy.

Agents to Avoid

Intravenous ketamine should be avoided given inconsistent efficacy (0-78%). Intravenous glycopyrrolate as monotherapy appears ineffective and should not be used.

Strengths and limitations of this review

Strengths

This systematic review represents the first comprehensive synthesis of evidence on the management of intraoperative erection, employing a rigorous methodology consistent with PRISMA 2020 guidelines. The search strategy was comprehensive, spanning multiple databases and supplementary sources over a 40-year period. Quality assessment using validated JBI tools was performed for all included studies. The multicentre, international nature of the included studies enhances generalisability of findings across different clinical settings. *Limitations*

The evidence base consists entirely of low-level evidence (case reports and case series) without comparative studies or randomised trials, limiting the strength of conclusions that can be drawn. Significant heterogeneity in interventions, dosing, and outcome reporting precluded meta-analysis. Quality assessment revealed methodological deficiencies in many studies, including inadequate demographic reporting and lack of consecutive patient inclusion. Publication bias likely affects this literature, with unsuccessful or complicated cases potentially underreported. Long-term follow-up data are absent, precluding assessment of potential delayed complications. The rarity of intraoperative erection made it impossible to calculate precise incidence rates or identify patient-specific risk factors.

## Conclusions

Intraoperative erection remains a rare but clinically significant complication of endourological surgery that can impair surgical access and delay procedures. While the evidence base consists entirely of low-level studies (case reports and case series), the consistency of outcomes across multiple independent reports from diverse geographical settings provides reasonable confidence in these findings. Based on the available evidence, intracavernosal injection of phenylephrine or ephedrine should be considered first-line, with intravenous sympathomimetics and dorsal nerve block serving as alternative or second-line options depending on clinical circumstances and available expertise.

The rarity of this complication and the ethical challenges of conducting controlled trials in the acute intraoperative setting make prospective comparative studies difficult but not impossible. Multicentre collaborative efforts to develop standardised protocols, establish registries, and conduct comparative effectiveness research would strengthen the evidence base and optimise management of this uncommon but important clinical scenario.

Until higher-level evidence becomes available, clinicians should be familiar with the techniques and relative merits of intracavernosal sympathomimetic injection as the most evidence-supported intervention for managing intraoperative erection during endourological procedures. Critically, given the unforeseen yet clinically significant nature of intraoperative erection and its management, it is important that routine pre-operative consenting discusses both the possibility of this event and the need for urgent pharmacological intervention, specifically, intracavernosal injection, so that patients are fully informed and consent to all aspects of their perioperative care.
